# Association Between Cholesterol–HDL–Glucose Index and Stroke Risk in Middle-Aged and Elderly Adults Without Diabetes: Evidence From Two National Cohort Studies

**DOI:** 10.14740/cr2258

**Published:** 2026-08-31

**Authors:** Yu Jing, Ben Lin, Chen Yang Ge, Bin Lu

**Affiliations:** a Department of Endocrinology, Huadong Hospital, Fudan University, Shanghai 200040, China; b Shanghai Key Laboratory of Clinical Geriatric Medicine, Huadong Hospital, Fudan University, Shanghai 200040, China; c Shanghai Institute of Geriatrics and Gerontology, Huadong Hospital, Fudan University, Shanghai 200040, China; d Department of Neurosurgery, Huashan Hospital, Fudan University, Shanghai 200040, China; e Shanghai Medical College, Fudan University, Shanghai 200032, China; f Yu Jing and Ben Lin contributed equally to this work.

**Keywords:** Stroke, Cholesterol–HDL–glucose index, Risk factors, CHARLS, NHANES

## Abstract

**Background:**

The cholesterol–HDL–glucose (CHG) index has emerged as a novel indicator for metabolic disorders, but its association with stroke risk, especially in nondiabetic individuals, remains unclear. This study aimed to investigate the relationship between the CHG index and stroke risk in middle-aged and elderly adults without diabetes.

**Methods:**

Data were derived from two cohorts: the China Health and Retirement Longitudinal Study (CHARLS) database (2011–2020) and the National Health and Nutrition Examination Survey (NHANES) database (2011–2018), respectively. The incidence of stroke events was the primary outcome. Cox proportional hazard regression models and restricted cubic spline (RCS) analysis were employed to examine the association between the CHG index and the risk of stroke. Receiver operating characteristic (ROC) curve was established to assess the diagnostic performance of the CHG index. Mediation analysis was conducted to reveal potential mediators of this association. Sensitivity and subgroup analyses were used to validate the robustness of the main findings.

**Results:**

In CHARLS cohort, a total of 651 participants (n = 8,376, 7.77%) developed stroke during the 9.0 years follow-up period. The multivariable adjusted hazard ratios for participants in the quartile 2 to quartile 4 groups compared with those in the quartile 1 group were 1.378 (95% confidence interval (CI) 1.073–1.771), 1.447 (95% CI 1.137–1.842), and 1.463 (95% CI 1.147–1.868), respectively. RCS analysis demonstrated a significant linear relationship between the CHG index and stroke risk (all P for nonlinear > 0.05). The ROC curve revealed that the CHG index had a comparable predictive performance with the triglyceride–glucose (TyG) index. Mediation analysis showed that glycated hemoglobin A1c (HbA1c) mediated 13.33% of the association between the CHG index and the stroke. Subgroup and sensitivity analyses further confirmed the robustness of the main findings, showing consistent results across different demographic and clinical groups. Additionally, analyses of the NHANES database (n = 5,057) indicated a significant positive correlation between the CHG index and stroke risk, as well as a notable predictive capacity of the CHG index for stroke.

**Conclusions:**

These findings suggest that the CHG index may serve as a novel and promising indicator for stroke.

## Introduction

Stroke remains a major cause of global mortality and long-term disability, imposing significant economic burdens on families and society [1]. The 2021 Global Burden of Disease Study (GBD) reported that the absolute number of stroke cases increased substantially from 1990 to 2021 [1]. In China, an estimated 17.8 million adults had suffered from a stroke in 2020, and 2.3 million died as a consequence [2]. Beyond this immense epidemiological burden, it should be emphasized that stroke is a highly heterogeneous disease. In clinical studies, it is extremely necessary to properly differentiate the various stroke subtypes, including atherothrombotic infarct, cardioembolic stroke, lacunar infarct, infarct of unusual etiology, and essential cerebral infarct. This differentiation is of great clinical interest due to the significant impact of stroke subtypes on the distribution of risk factors, stroke severity, and patient outcomes [3]. Despite substantial improvements in primary prevention and treatment, the occurrence of stroke continues to rise [4]. Therefore, it is vital to identify modifiable risk factors to mitigate stroke incidence and alleviate financial strains.

Insulin resistance (IR), characterized by impaired sensitivity of target organs to insulin, has emerged as an independent risk factor for stroke [5, 6]. Emerging evidence has illustrated the intricate relationship between IR and stroke, as IR can lead to stroke by promoting inflammation, oxidative stress, and neuronal damage [7]. Traditional methods for assessing IR mainly include the hyperinsulinemic-euglycemic clamp (HEC) and the homeostasis model assessment of IR (HOMA-IR) [8, 9]. These methods are not suitable for clinical and epidemiological research due to its complexity, high cost, and invasiveness. Consequently, various surrogate indexes of IR, such as the triglyceride–glucose (TyG) index and TyG-related index have received increasing attention [10–12]. Among these indices, the cholesterol–HDL–glucose (CHG) index has been regarded as a novel biomarker. This index was initially proposed by Mansoori et al for diagnosing type 2 diabetes mellitus (T2DM), which exhibits higher efficiency value than TyG index [13]. Thereafter, Mo et al reported its role in predicting the cardiovascular disease (CVD) risk, as an elevated CHG index is significantly correlated with an increased risk of CVD [14]. Besides, Dong et al identified a significant association between the CHG index and the rapid kidney function decline in patients with cardiovascular-kidney-metabolic (CKM) syndrome [15]. Previous studies have shown that the CHG index is positively associated with stroke risk among individuals with and without diabetes [16]. Given that adverse lifestyle behaviors and environmental exposures commonly associated with diabetes may confound study outcomes, the present study restricted its cohort to individuals without diabetes to mitigate potential bias, and investigate the relationship by analyzing two national cohorts.

Thus, we used data from the China Health and Retirement Longitudinal Study (CHARLS) and National Health and Nutrition Examination Survey (NHANES) to explore the association between the CHG index and the risk of stroke in middle-aged and elderly adults without diabetes. The findings of this study may provide new insights into the treatment and prevention of stroke, as well as evidence into the application of the CHG index in clinical practice.

## Materials and Methods

### Study design and participants

Our research used data from the CHARLS waves conducted in 2011 and 2020 to perform a prospective study. CHARLS represents a nationally representative cohort of middle-aged and elderly individuals aged 45 and above. The detailed study design and enrollment standards have been outlined in prior publications [17]. Briefly, the baseline survey began in 2011 (wave 1), enrolling 17,708 participants from 150 districts or counties across 28 provinces in China. Follow-up assessments were conducted every 2 to 3 years, with data collected via in-person interviews. At present, the study has completed four waves follow-up (wave 2 in 2013, wave 3 in 2015, wave 4 in 2018, and wave 5 in 2020). The study obtained approval from the Biomedical Ethics Review Board of Peking University (IRB00001052-11015). All participants provided written informed consent before enrolling in the study.

Besides, we also used data from NHANES 2011–2018 to conduct a cross-sectional study. NHANES is a nationally representative survey mainly focused on the health and nutritional conditions of noninstitutionalized civilians in the United States (US). The study employs a sophisticated multistage probability sampling design to ensure the selection of nationally representative samples. The study obtained approval from the Ethics Review Board of the National Center for Health Statistics, and written informed consent was obtained from all participants prior to data collection.

**Figure 1:**
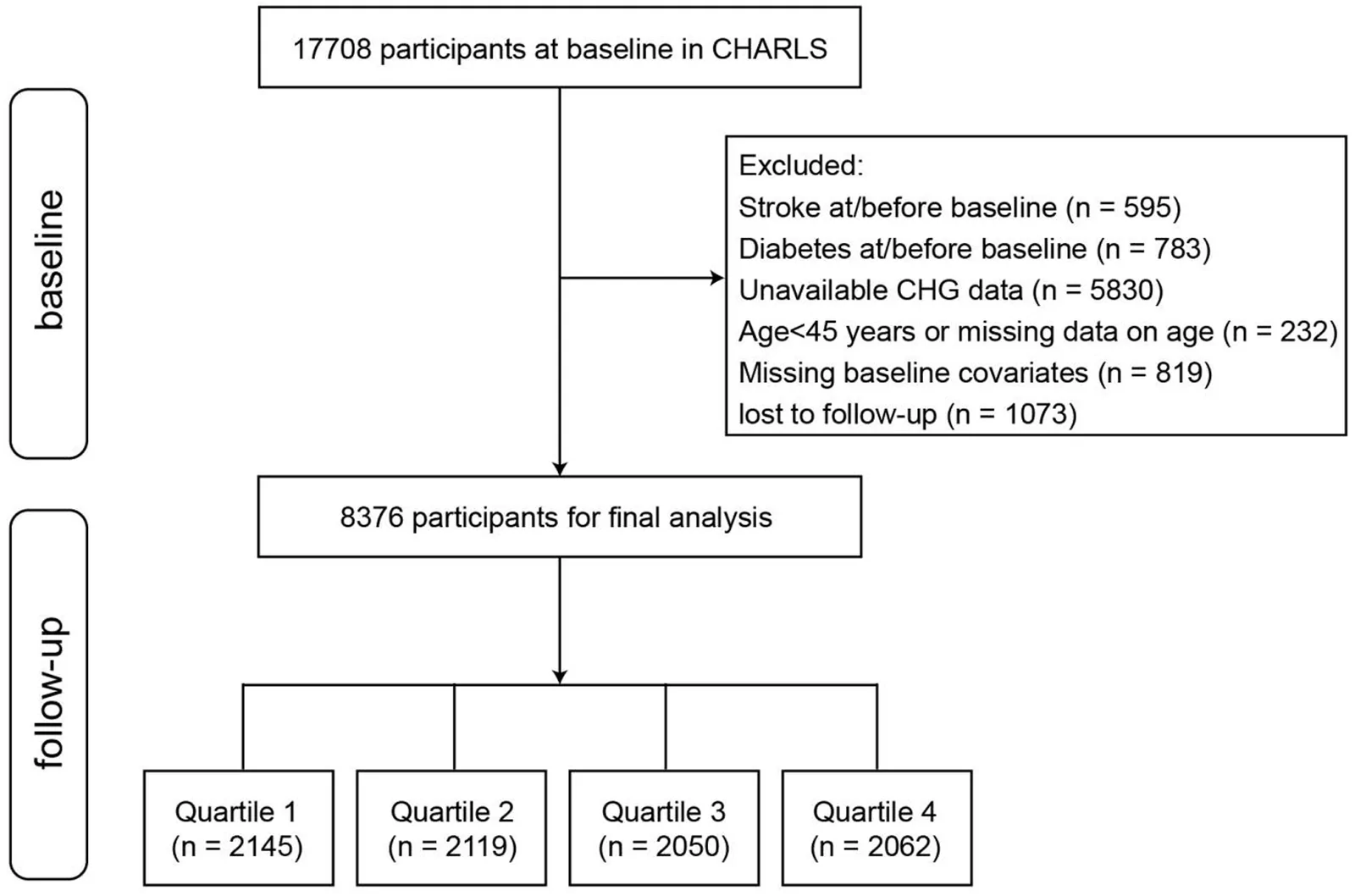
Flowchart of the study participants.

In CHARLS cohort, the inclusion criteria were participants with available CHG index data. We excluded 9,332 participants following criteria: (1) those younger than 45 years or lacking age information; (2) individuals diagnosed with stroke or missing stroke diagnosis data at baseline; (3) individuals who were lost to follow-up; (4) those missing baseline covariates; (5) individuals with incomplete data on fasting blood glucose (FBG), total cholesterol (TC), and low-density lipoprotein (LDL); (6) individuals with diabetes or missing diabetes information. Finally, a total of 8,376 participants were eligible for subsequent analyses. The detailed screening process is illustrated in Figure 1.

To ensure consistency and data quality across cohorts, the inclusion and exclusion criteria in NHANES cohort were similar to that of the CHARLS cohort. Participants were included if they were aged 45 years or elder, and had complete data on stroke, diabetes, FBG, TC, and LDL. The cohort initially comprised 39,156 participants from 2011 to 2018. The final analyses included 5,057 participants stratified into four quartiles according to the CHG index.

### Data collection and measurement

Trained interviewers gathered sociodemographic and health-related information via standardized questionnaires. Sociodemographic characteristics included age, gender, marital status (married or others), residence (rural or urban), and educational level (below primary, primary school, middle school, or high school or above). Height, weight, and waist circumstance (WC) were also measured. Health-related data included self-reported smoking and drinking habits, and physician diagnosed conditions such as hypertension, cancer, dyslipidemia, heart disease, and kidney disease. Additionally, fasting venous blood samples were obtained and analyzed for biochemical parameters. Serum triglycerides (TG), TC, low-density lipoprotein cholesterol (LDL-C), high-density lipoprotein cholesterol (HDL-C), FBG, and glycated hemoglobin (HbA1c) were measured based on an enzymatic colorimetric test. The coefficient of variation for all blood biomarker measurements is less than 5%, which ensures the high reliability of the results.

Blood pressure was determined as the average of three-time measurements taken while sitting after 5 min rest. Hypertension was defined as follows: a self-reported diagnosis of hypertension, and/or any use of antihypertensive drugs, and/or an average systolic/diastolic blood pressure (SBP/DBP) ≥ 140/90 mm Hg. Dyslipidemia was defined as self-reported dyslipidemia history, and/or current use of lipid-lowering treatment, and/or TC ≥ 240 mg/dL, TG ≥ 150 mg/dL, LDL-C ≥ 160 mg/dL, HDL-C < 40 mg/dL. Diabetes was defined based on a self-reported physician diagnosis, and/or use of antidiabetic medications, and/or FBG ≥ 126 mg/dL, and/or an HbA1c level ≥ 6.5%. Body mass index (BMI) was calculated using the formula: BMI (kg/m^2^) = body mass (kg)/height^2^ (m^2^).

### Assessment of CHG index

The formula for calculating the CHG index was as follows: CHG index = Ln (TC (mg/dL) × FBG (mg/dL)/2 × HDL (mg/dL)).

### Assessment of stroke events

The primary outcome of this study was the incidence of stroke. In CHARLS cohort, stroke was determined via the standardized questionnaire, which ask participants: “Have you been diagnosed with stroke by a doctor?” or “Have you been diagnosed with stroke by a doctor since the last follow-up visit?”. The time of stroke events was established based on participants’ responses to specific questions: “When was the stroke first diagnosed or known by yourself?” or “When was your most recent stroke?”. Each participant was followed up from baseline (2011) until the occurrence of the stroke or the most recent survey (2020), whichever came first. In NHANES cohort, stroke was defined by self-reported previous diagnosis by a physician during in-person interview. Participants who answered “yes” to the question, “Have you ever been told by a physician or a health professional that you had stroke?”, were classified as having experienced a stroke.

### Missing data processing

In order to reduce bias resulting from missing variables, multiple imputation was used to estimate the missing values, assuming the data were missing at random (MAR).

### Statistical analysis

Continuous variables that followed normal distribution were expressed as mean ± standard deviation (SD), while those with skewed distribution were presented as median (interquartile range (IQR)). To compare baseline data for normally and skewed distributed variables, one-way analysis of variance (ANOVA) and the Kruskal–Wallis test were employed, respectively. Categorical variables were presented as frequencies and percentages, with differences assessed using Chi-square test or Fisher’s exact test. All participants were categorized into four groups according to the quartiles of the CHG index. The CHG index was also analyzed as a continuous variable to enhance the robustness and reliability of the findings. In addition, for the NHANES cohort, all analyses adhered to NHANES analytic guidelines by incorporating appropriate survey weights, stratification, and primary sampling unit variables.

To examine the relationship between the CHG index and stroke risk, Cox proportional hazard regression models were utilized to determine the hazard ratio (HR) along with a 95% confidence interval (CI). The proportional hazard assumption was verified using Schoenfeld residuals, revealing no indications of potential violations. The CHG index was incorporated into the models both as continuous and categorical variables. Three distinct models were assessed. Model 1 was unadjusted. Model 2 included adjustments for age and gender. Model 3 made further adjustment for marital status, residence, education, smoking status, drinking status, and hypertension. All adjusted variables were analyzed for collinearity, and no significant multicollinearity was detected.

Additionally, RCS regression analysis, which included multivariable-adjusted Cox regression, was employed to explore the linear or nonlinear relationship between the CHG index and the risk of stroke. The receiver operating characteristic (ROC) curve was established to assess the diagnostic value of the CHG index on the incidence of stroke. The area under the ROC curve (AUC) was calculated to quantify the predictive power of the CHG index for stroke occurrence.

Mediation analysis was employed to investigate whether HbA1c mediated the association between the CHG index and the stroke. The average causal mediation effect (ACME), average direct effect (ADE), and the proportion mediated were calculated. The corresponding significance was evaluated using a bootstrap method involving 1,000 iterations.

Subgroup and interaction analyses were utilized to examine the impact of the CHG index on stroke risk in several subgroups, including age (< 60 years/≥ 60 years), gender (male/female), residence (rural/urban), educational level (below primary, primary school, middle school, or high school or above), smoking (yes/no), drinking (yes/no), and hypertension (yes/no).

Several sensitivity analyses were performed to assess the robustness of the main findings. In sensitivity analysis 1, participants with any missing data were removed. In sensitivity analysis 2, participants who had used lipid-lowering drugs at baseline were excluded from the analysis. In sensitivity analysis 3, participants who were receiving antihypertensive drugs at baseline were removed from the analysis. In sensitivity analysis 4, participants with a follow-up period of less than 2 years were excluded.

Lastly, to elucidate the relationship between the CHG index and stroke incidence in NHANES cohort, we developed three weighted logistic regression models. Model 1 was unadjusted. Model 2 adjusted for age and gender. Model 3 adjusted for age, gender, marital status, education, smoking status, drinking status, and hypertension. Odds ratio (OR) and 95% CI were calculated to quantify the associations within the three models.

All statistical analyses were conducted using IBM-SPSS (version 26.0, Chicago, IL, USA), R software (version 4.5.0, Vienna, Austria), and GraphPad Prism (version 9.0). A two-tailed P value < 0.05 was considered statistically significant.

## Results

### Baseline characteristics of study participants

**Figure 2:**
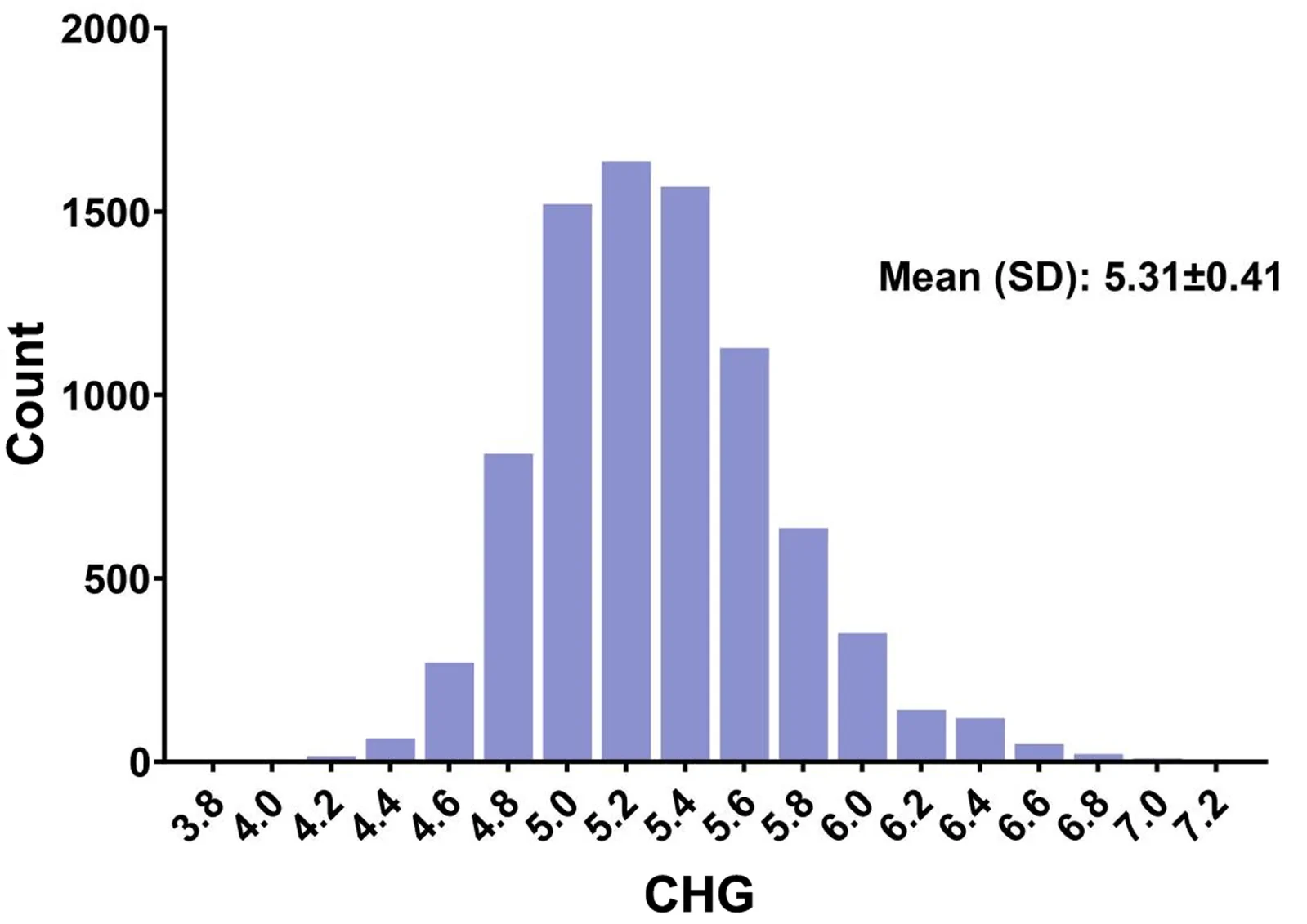
Distribution of the CHG index. CHG index: cholesterol–HDL–glucose index.

A total of 8,376 participants were included in this study, with an average age of 57.00 years and 44.72% being male. Participants were categorized into four subgroups according to the quartiles of the CHG index. The baseline characteristics of the participants are summarized in Table 1, and the distribution of the CHG index is shown in Figure 2. Compared with participants in the lowest quartile of the CHG index, those in higher quartile were more likely to be elder, married, living in urban areas, and had lower rates of drinking. Among participants in higher CHG index quartile, the prevalence of chronic disease (hypertension, dyslipidemia, and heart disease) and the usage of drugs were higher. Additionally, these groups exhibited higher levels of WC, BMI, SBP, DBP, TC, TG, LDL-C, HbA1c, and FBG, while HDL-C levels were lower.

**Table 1:** Baseline Characteristics of Participants

Characteristics	Overall	Quartiles of CHG				P value
		Q1	Q2	Q3	Q4	
Number of participants	8,376	2,145	2,119	2,050	2,062	
Age, years	57.00 (51.00–63.00)	57.00 (50.00–63.00)	57.00 (51.00–63.00)	57.00 (51.00–63.00)	58.00 (52.00–64.00)	0.009
Gender, n (%)						0.088
Male	3,746 (44.72%)	1,008 (46.99%)	922 (43.51%)	914 (44.59%)	902 (43.74%)	
Female	4,630 (55.28%)	1,137 (53.01%)	1,197 (56.49%)	1,136 (55.41%)	1,160 (56.26%)	
Marital status, n (%)						0.026
Married	8,239 (98.36%)	2,095 (97.67%)	2,090 (98.63%)	2,018 (98.44%)	2,036 (98.74%)	
Others	137 (1.64%)	50 (2.33%)	29 (1.37%)	32 (1.56%)	26 (1.26%)	
Residence, n (%)						< 0.001
Rural	7,008 (83.67%)	1,889 (88.07%)	1,806 (85.23%)	1,688 (82.34%)	1,625 (78.81%)	
Urban	1368 (16.33%)	256 (11.93%)	313 (14.77%)	362 (17.66%)	437 (21.19%)	
Education, n (%)						0.078
Below primary school	3,907 (46.64%)	1,066 (49.70%)	975 (46.01%)	948 (46.24%)	918 (44.52%)	
Primary school	1,809 (21.60%)	449 (20.93%)	477 (22.51%)	434 (21.17%)	449 (21.77%)	
Middle school	1,758 (20.99%)	417 (19.44%)	449 (21.19%)	438 (21.37%)	454 (22.02%)	
High school or above	902 (10.77%)	213 (9.93%)	218 (10.29%)	230 (11.22%)	241 (11.69%)	
Smoking, n (%)	3,116 (37.20%)	845 (39.39%)	769 (36.29%)	762 (37.17%)	740 (35.89%)	0.082
Drinking, n (%)	3,178 (37.94%)	905 (42.19%)	787 (37.14%)	753 (36.73%)	733 (35.55%)	< 0.001
Hypertension, n (%)	2,705 (32.29%)	528 (24.62%)	600 (28.32%)	700 (34.15%)	877 (42.53%)	< 0.001
Dyslipidemia, n (%)	3,983 (47.55%)	287 (13.38%)	619 (29.21%)	1,206 (58.83%)	1,871 (90.74%)	< 0.001
Cancer, n (%)	80 (0.96%)	18 (0.84%)	22 (1.04%)	19 (0.93%)	21 (1.02%)	0.904
Heart disease, n (%)	914 (10.91%)	198 (9.23%)	221 (10.43%)	219 (10.68%)	276 (13.39%)	< 0.001
Kidney disease, n (%)	531 (6.34%)	156 (7.27%)	134 (6.32%)	115 (5.61%)	126 (6.11%)	0.160
Medication, n (%)						
Lipid-lowering	395 (4.72%)	40 (1.86%)	80 (3.78%)	90 (4.39%)	185 (8.97%)	0.002
Antihypertensive	1,455 (17.37%)	259 (12.07%)	292 (13.78%)	376 (18.34%)	528 (25.61%)	0.011
WC, cm	84.20 (77.30–91.50)	79.20 (73.60–86.00)	82.40 (76.40–89.00)	86.40 (79.50–93.07)	90.00 (83.00–97.00)	< 0.001
BMI, kg/m^2^	23.17 (20.90–25.81)	21.50 (19.73–23.77)	22.64 (20.62–24.84)	23.86 (21.56–26.30)	24.99 (22.70–27.55)	< 0.001
SBP, mm Hg	125.67 (113.67–140.33)	122.00 (111.00–136.67)	123.67 (112.33–138.33)	127.33 (115.00–141.33)	130.50 (118.67–145.00)	< 0.001
DBP, mm Hg	74.33 (67.00–83.00)	72.33 (65.00–81.00)	73.33 (66.00–82.00)	75.00 (68.33–83.33)	77.33 (70.00–85.33)	< 0.001
TC, mg/dL	190.21 (167.40–214.95)	173.20 (155.03–195.62)	186.34 (165.46–207.60)	195.43 (173.20–216.88)	209.15 (184.79–239.31)	< 0.001
TG, mg/dL	105.32 (75.22–153.99)	72.57 (57.53–93.81)	92.04 (71.68–120.36)	121.25 (92.04–157.53)	178.77 (126.78–249.57)	< 0.001
HDL-C, mg/dL	49.48 (40.59–59.92)	63.02 (54.90–73.45)	52.96 (46.39–60.70)	45.23 (39.82–52.19)	37.89 (32.47–44.46)	< 0.001
LDL-C, mg/dL	114.82 (94.33–137.63)	96.26 (81.96–112.89)	115.21 (97.81–132.60)	124.87 (104.87–144.88)	128.74 (103.32–156.57)	< 0.001
HbA1c, %	5.10 (4.90–5.40)	5.00 (4.80–5.30)	5.10 (4.90–5.40)	5.20 (4.90–5.40)	5.30 (5.00–5.70)	< 0.001
FBG, mg/dL	100.06 (92.27–110.32)	94.32 (87.48–100.98)	99.72 (93.69–106.47)	103.50 (96.84–112.14)	106.88 (99.48–118.27)	< 0.001
CHG index	5.28 (5.03–5.55)	4.87 (4.75–4.96)	5.16 (5.10–5.23)	5.41 (5.34–5.48)	5.78 (5.65–5.98)	0.009
Stroke, n (%)	651 (7.77%)	115 (5.36%)	147 (6.94%)	179 (8.73%)	210 (10.18%)	< 0.001

Data are presented as median (interquartile range) or n (%). BMI: body mass index; CHG index: cholesterol–HDL–glucose index; DBP: diastolic blood pressure; FBG: fasting blood glucose; HbA1c: hemoglobin A1c; HDL-C: high-density lipoprotein cholesterol; LDL-C: low-density lipoprotein cholesterol; SBP: systolic blood pressure; TC: total cholesterol; TG: triglyceride; WC: waist circumference.

### Association and dose–response relationship between the CHG index and stroke risk

Over a maximum follow-up duration of 9.0 years, 651 new cases of stroke were recorded, accounting for 7.77% of the participants. The percentages of stroke for participants in quartiles 1 to 4 were 5.36%, 6.94%, 8.73%, and 10.18%, respectively.

To evaluate the relationship between the CHG index level and the risk of stroke, Cox proportional hazard regression models were conducted. Table 2 exhibits the results from three Cox regression models. When considered as a continuous variable, each 1-unit increase in the CHG index was associated with a higher risk of stroke in unadjusted model 1 (HR 1.258, 95% CI 1.065–1.486), model 2 (HR 1.264, 95% CI 1.070–1.493), and the fully adjusted model 3 (HR 1.255, 95% CI 1.038–1.518). Similar trends were observed in the analysis stratified by the CHG index quartiles. Compared with the quartile 1 group, the multivariable adjusted HRs for quartiles 2 to 4 in model 3 were as follows: 1.378 (95% CI 1.073–1.771), 1.447 (95% CI 1.137–1.842), and 1.463 (95% CI 1.147–1.868), respectively. Additionally, a significant trend of increasing stroke risk across the CHG index quartiles was observed in all three models (all P value < 0.05).

**Table 2:** Association of CHG Index and Risk of Stroke

	Case	Model 1		Model 2		Model 3	
		HR (95% CI)	P value	HR (95% CI)	P value	HR (95% CI)	P value
CHG (per 1-unit)	651	1.258 (1.065–1.486)	0.007	1.264 (1.070–1.493)	0.006	1.255 (1.038–1.518)	0.019
CHG quartile							
Q1	115	Ref		Ref		Ref	
Q2	147	1.358 (1.063–1.734)	0.014	1.366 (1.070–1.744)	0.012	1.378 (1.073–1.771)	0.012
Q3	179	1.414 (1.118–1.789)	0.004	1.417 (1.120–1.793)	0.004	1.447 (1.137–1.842)	0.003
Q4	210	1.473 (1.173–1.851)	< 0.001	1.481 (1.179–1.861)	< 0.001	1.463 (1.147–1.868)	0.002

Model 1: unadjusted. Model 2: adjusted for age and gender. Model 3: adjusted for age, gender, marital status, residence, education, smoking status, drinking status, and hypertension. CHG index: cholesterol–HDL–glucose index; CI: confidence interval; HR: hazard ratio.

**Figure 3:**
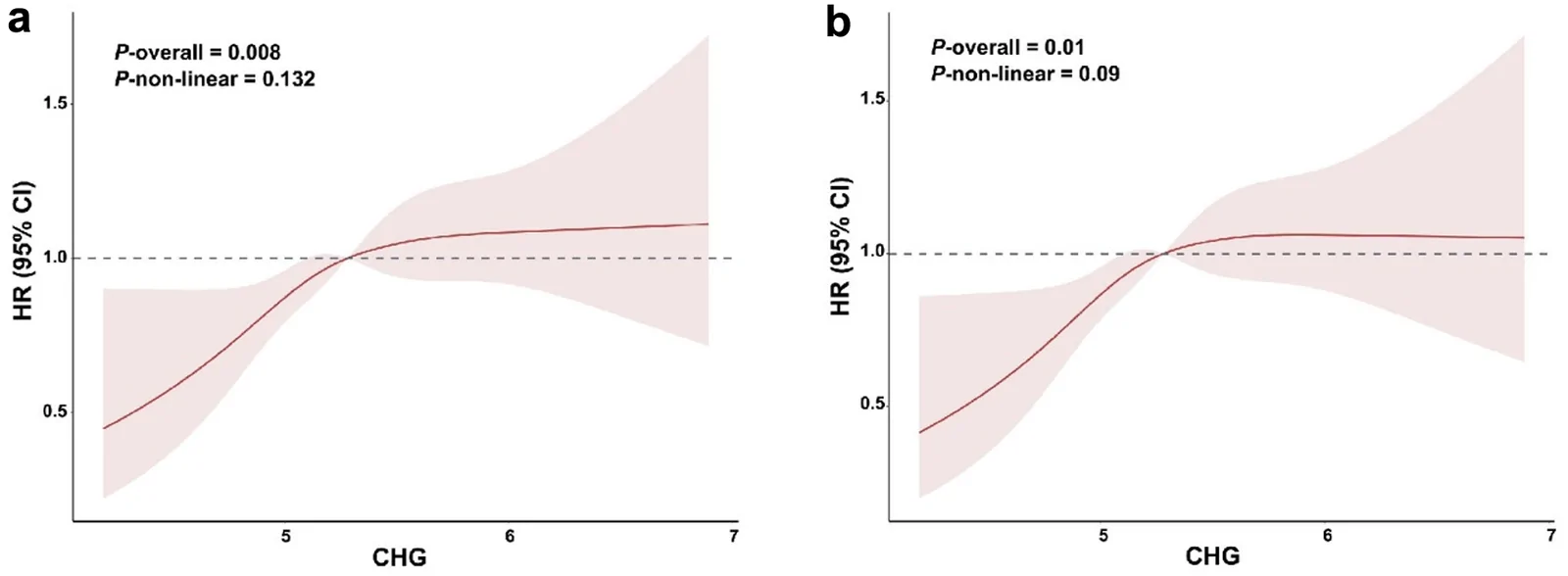
Restricted cubic spline models analyze the relationship between the CHG index and stroke incidence. (a) Unadjusted Model. (b) Adjusted for age, gender, marital status, residence, education, smoking status, drinking status, and hypertension. CHG index: cholesterol–HDL–glucose index.

**Figure 4:**
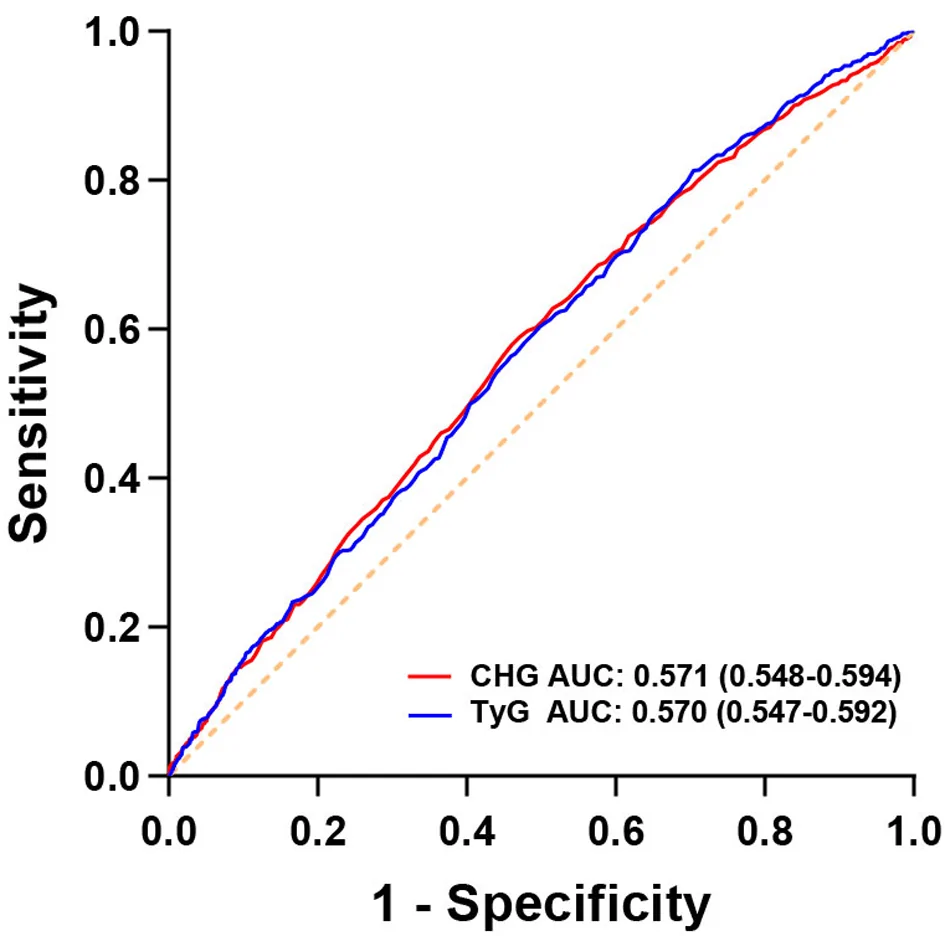
Receiver operating characteristic curve for the CHG index and the TyG index in predicting stroke incidence. CHG index: cholesterol–HDL–glucose index; TyG index: triglyceride–glucose index.

The dose–response curves for the association between the CHG index and stroke risk are presented in Figure 3. RCS analysis indicated a significant linear relationship between the CHG index and the incidence of stroke, both with or without adjusting for covariates (all P for nonlinear > 0.05). The ROC curve (Fig. 4) suggested that the predictive ability of the CHG index for stroke incidence (AUC 0.571, 95% CI 0.548–0.594) was comparable to that of the traditional TyG index (AUC 0.570, 95% CI 0.547–0.592). The results indicate that the CHG index exhibits a modest predictive value for stroke.

### Mediation analysis

**Figure 5:**
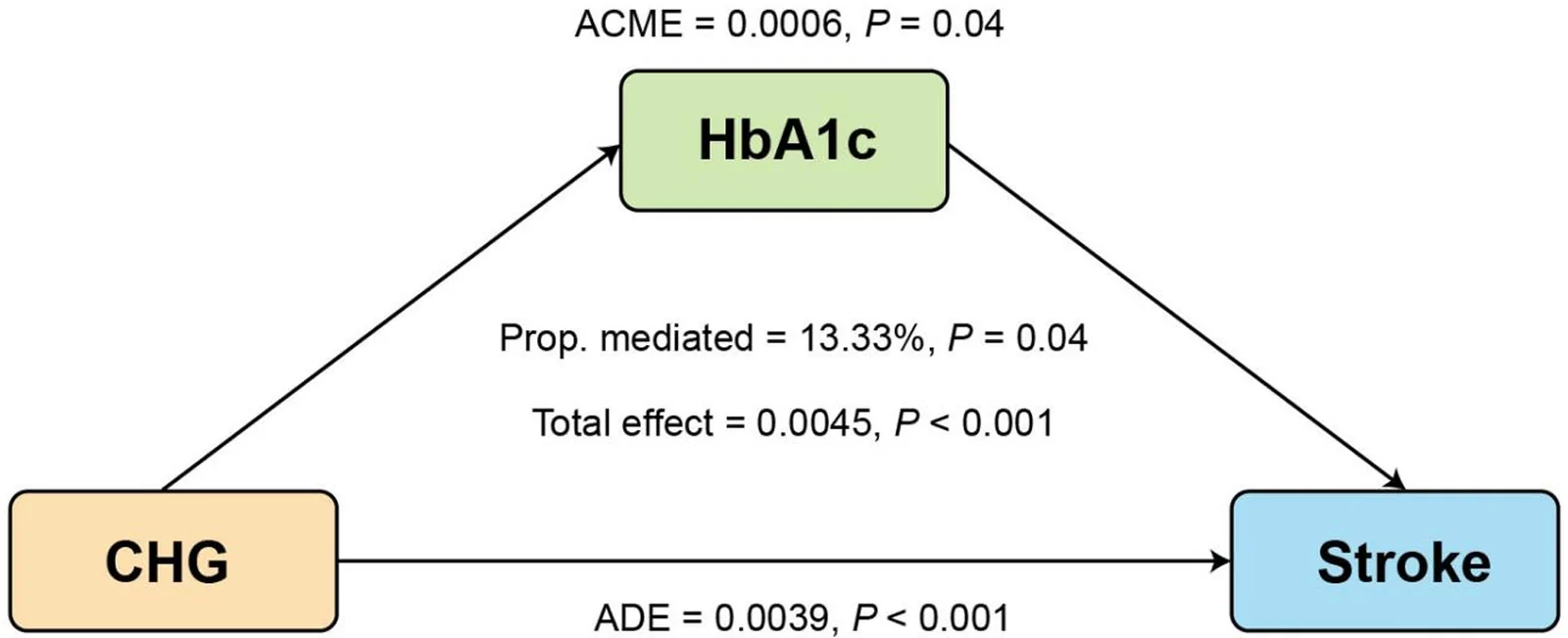
Mediation analysis of HbA1c in the association between the CHG index and the stroke risk. Adjusted for age, gender, marital status, residence, education, smoking status, drinking status, and hypertension. CHG index: cholesterol–HDL–glucose index.

To identify the potential mediating factors between the CHG index and the stroke, we performed mediation analysis. The result demonstrated that HbA1c significantly mediated the association between the CHG index and the stroke (Fig. 5). In the fully adjusted model, the overall effect proved to be significant (estimate = 0.0045, P < 0.001), showing an ADE of 0.0039 (P < 0.001) and an ACME of 0.0006 (P < 0.05). The proportion mediated by HbA1c was 13.33%. The result indicates that the association between the CHG index and incident stroke is partially mediated by HbA1c levels.

### Subgroup and sensitivity analyses

To further analyze the connection between the CHG index and the risk of stroke, we performed subgroup and interaction analyses according to factors such as age, gender, residence, educational level, smoking, drinking, and hypertension.

**Figure 6:**
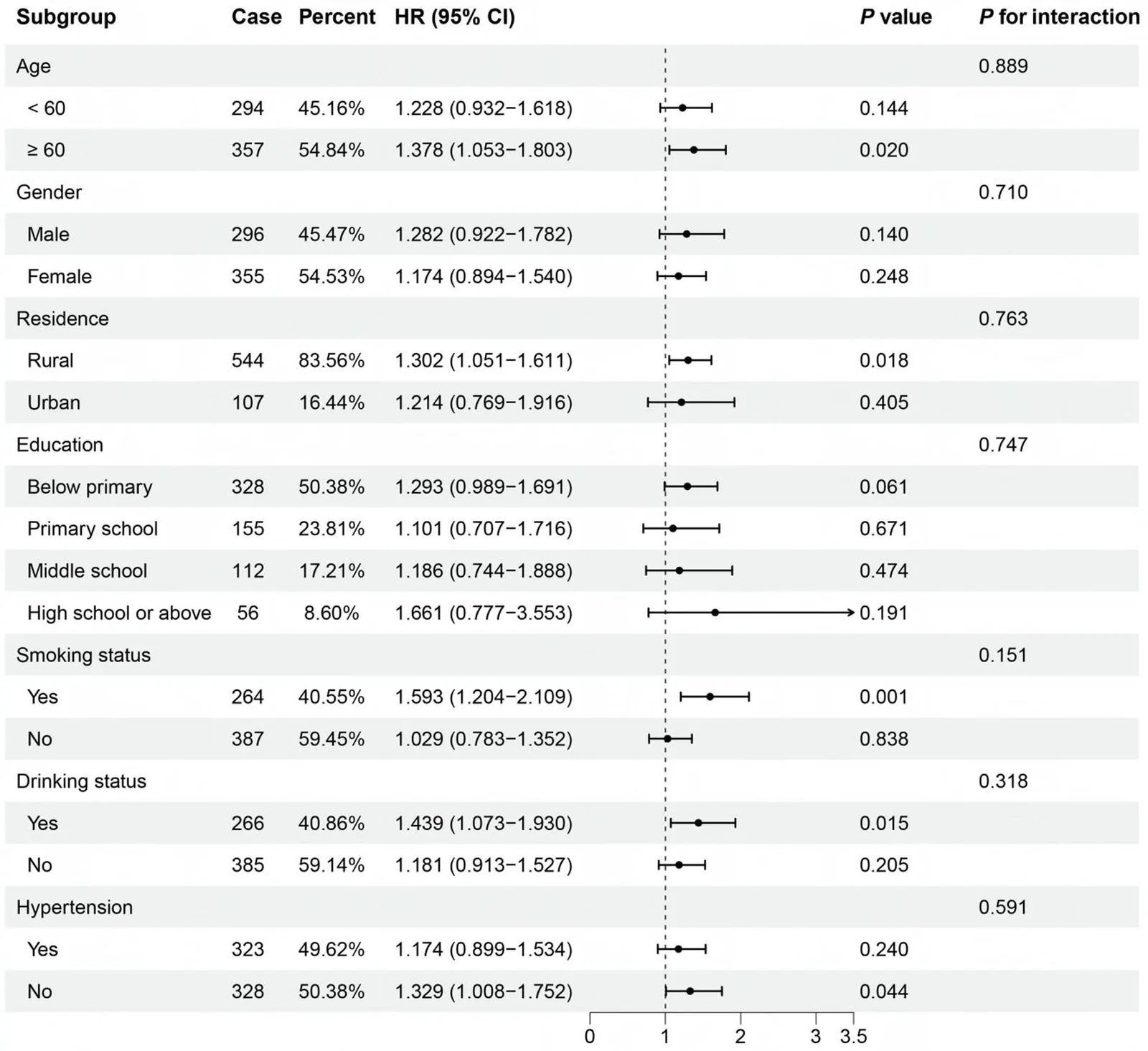
Subgroup and interaction analyses of the association between the CHG index and the risk of stroke. Adjusted based on model 3, with the stratification variable itself excluded from adjustment. CHG index: cholesterol–HDL–glucose index.

As illustrated in Figure 6, the relationship between the CHG index and the risk of stroke remained consistent with the main findings across most subgroups, with no notable interactions detected. This indicates that the impact of the CHG index on the risk of developing stroke is consistent across diverse demographic and clinical categories.

To ensure the robustness of the main findings, several sensitivity analyses were carried out. Initially, the results remained consistent with the primary analysis after excluding participants with any missing data (Supplementary Material 1, cr.elmerpub.com). Second, this association also remained unchanged after removing participants receiving lipid-lowering treatments or antihypertensive therapies at baseline (Supplementary Materials 2 and 3, cr.elmerpub.com). Furthermore, the association between the CHG index and stroke risk was not substantially altered even after removing participants whose follow-up duration was less than 2 years (Supplementary Material 4, cr.elmerpub.com).

### Association of the CHG index with incident stroke in NHANES cohort

To confirm the reliability of the main findings, we utilized NHANES cohort, a nationally representative database of US population, for external validation. A total of 5,057 participants were included in the analysis, with a weighted average age of 61.57 years. Baseline characteristics of the selected participants are summarized in Supplementary Material 5 (cr.elmerpub.com). We performed several weighted logistic regression analyses to detect the association between the CHG index and stroke incidence. As shown in Supplementary Material 6 (cr.elmerpub.com), a higher CHG index was positively associated with increased stroke incidence in unadjusted model 1 (OR 2.853, 95% CI 1.851–4.398), model 2 (OR 3.497, 95% CI 2.281–5.361), and model 3 (OR 2.610, 95% CI 1.729–3.937). The results remain consistent when the CHG index was considered as a categorical variable. After adjustment for potential confounders in model 3, the ORs with 95% CIs for stroke in quartiles 2 to 4 were 1.738 (95% CI 1.179–2.562), 1.901 (95% CI 1.234–2.931), and 2.467 (95% CI 1.685–3.613) compared to the lowest quartile, respectively. The ROC curve (Supplementary Material 7, cr.elmerpub.com) verified the predictive performance of the CHG index for diagnosing stroke (AUC = 0.561, 95% CI 0.539–0.584). Consistent with CHARLS results, the aforementioned findings reinforce the CHG index as a prognostic marker for stroke.

## Discussion

In this population-based cohort study, we firstly explored the association between the CHG index and stroke risk in CHARLS cohort, which encompass 8,376 Chinese nondiabetic individuals with a maximum follow-up duration of 9.0 years. Our results indicated a notable link between higher levels of the CHG index and an increased risk of stroke after adjusting for various confounding variables. Importantly, we discovered a linear relationship between the CHG index and stroke risk. And HbA1c was found to partially mediated the relationship between the CHG index and stroke. Moreover, both subgroup and sensitivity analyses consistently supported the primary findings. Apart from these, we also verified the findings by analyzing the NHANES cohort, a database representative of the US population. And the results confirmed the positive association between the CHG index and stroke incidence among individuals without diabetes, as well as the predictive capacity of the CHG index for stroke. Generally, these results propose that the CHG index is a promising diagnostic biomarker for early identification and prompt prevention of stroke.

The CHG index, which integrates TC, HDL, and FBG levels, is recently reported by Mansoori et al as a new index for diagnosing T2DM [13]. They discovered that in Iranian cohort, the CHG index had a lower sensitivity and higher specificity value compared to the well-established TyG index. Since then, accumulating evidence has supported the use of the CHG index as a reliable indicator for predicting T2DM and its related complications. Zhang et al further validated the predictive ability of the CHG index for T2DM within the US population [18]. Li et al verified the diagnostic role of the CHG index for T2DM in Chinese population [19]. Meanwhile, Catak et al assess the relationship between the CHG index and the presence of diabetic retinopathy and nephropathy in T2DM patients [20]. More recently, the application of the CHG index has expanded to nondiabetic populations. A retrospective study included patients with IgA nephropathy demonstrated that elevated CHG index is strongly associated with renal prognosis in these patients [21]. The CHG index has also been revealed to be an independent predictor for diagnosing hypertension, carotid plaque, and fatty liver disease [22, 23]. Additionally, Wang et al discovered the predictive value of the CHG index in assessing stroke risk [16]. Diabetic individuals may demonstrate diminished sensitivity to predictive indicators due to the influence of other risk factors, a situation often described as the ceiling effect [24]. Furthermore, identifying risk factors within nondiabetic populations could facilitate earlier interventions, thereby carrying profound implications for reducing the overall burden of disease. Given these considerations, we explored the association between the CHG index and stroke risk in individuals without diabetes utilizing data form two large population cohorts.

In CHARLS cohort, we observed that participants with higher CHG index levels were generally elder, at greater risk of suffering comorbid chronic diseases, and had a higher ratio of using medications compared to those with lower CHG index levels. This phenomenon is commonly acceptable. Due to aging, elderly participants are prone to have hyperglycemia and dyslipidemia; consequently, the CHG index, which considers both lipid factors and blood glucose levels, tends to be higher in these population. After adjusting for various variables, the Cox regression analysis demonstrated that an elevated CHG index level was independently associated with an increased risk of stroke in middle-aged and elderly adults without diabetes. Compared with the quartile 1 group, each 1-unit increase in quartile 4 group was associated with a 46.3% increase in stroke risk. Consistent with our results, multiple studies have revealed that nontraditional lipid profiles, including TC and HDL, are significantly associated with the stroke incidence [25–27]. The TC/HDL ratio has been widely recognized as a surrogate marker for evaluating dyslipidemia. Xiao et al have established a notable positive association between the TC/HDL ratio and stroke risk, particularly in middle-aged and elderly populations [28]. Tang et al also validated this correlation in ischemic stroke population [29]. The CHG index we examined takes into account not only lipid profiles but also blood glucose levels. Blood glucose has also been reported to be positively related to stroke risk [30]. Therefore, the CHG index may offer superior predictive values compared to the TC/HDL ratio. Apart from these, mediation analysis indicated that HbA1c partially mediated the relationship between the CHG index and the stroke. Besides, to mitigate the bias derived from some confounding factors, we conducted several subgroup and sensitivity analyses, including age, gender, residence, educational level, smoking, drinking, and hypertension, and the results remained consistent when these factors were excluded. Notably, it is reported that women differ from men in the distribution of risk factor, stroke subtype, stroke severity, and clinical outcome [31]. Our subgroup analysis showed that the association between the CHG index and stroke risk was consistent across both sexes. These findings suggest that, despite the well-established sex-specific disparities in stroke, the CHG index serves as a robust and consistent risk predictor for stroke across both sexes. In addition, the demographic and risk factors of stroke patients aged 85 years and older differ substantially from those of younger age groups [32]. Due to the limited sample size of this population in our study, we were unable to perform a subgroup analysis for this age group, which deserves further investigation in larger, age-specific cohorts.

Furthermore, our study showed a significant linear correlation between the CHG index and stroke risk. In alignment with our study, the CHG index was also reported to exhibit a positive linear correlation with the risk of CVD [14]. Though the CHG index showed a nonlinear relationship with T2DM [18], we speculate that this discrepancy may be attributed to the diversities of disease and the differences in inclusion criteria. Additionally, in the current study, ROC curve was also used to evaluate the CHG index for predicting stroke risk. The results demonstrated that the CHG index exhibits modest predictive ability, showing comparable predictive performance to the traditional TyG index. Apart from these, we verified the robustness of the main findings through analyzing the NHANES cohort. We also discovered a significant positive correlation between the CHG index and the incident stroke in US population, whereby a higher CHG index was associated with an increased stroke incidence. The ROC curve further confirmed the predictive capacity of the CHG index for stroke.

So far, previous studies have mainly focused on the relationship between the CHG index and diabetes. Though Mo et al investigated the correlation between the CHG index and CVD, the direct relationship between the CHG index and stroke, especially in nondiabetic individuals, is still unclear. Therefore, our study further expands the current understanding of the relationship between the CHG index and stroke in populations without diabetes, providing a simple and non-expensive tool for the detection and prevention of stroke.

The potential mechanisms linking the CHG index and stroke are likely to be multifaceted and not fully understood, but several plausible mechanisms may clarify this association. First, as a surrogate marker of IR, the CHG index level is positively linked to the risk of IR. The hyperinsulinemia leads to endothelial dysfunction, elevated pro-inflammatory cytokines secretion, and arterial stiffness, which collectively accelerate stroke occurrence [33, 34]. Besides, the CHG index also reflects multifaceted dysregulation in glucose and lipid metabolism, oxidative stress, and chronic inflammation. In this study, we identified that CHG index may contribute to the incidence of stroke by partially mediating the effects of HbA1c in human metabolism. HbA1c is a critical clinical biomarker that reflects recent glycemic status [35]. Either abnormal regulation in glucose or lipid metabolism may cause irreversible vascular damage, foam cell formation, and platelet coagulation [36, 37]. The accompanied oxidative stress also activates the PKC/NADPH oxidase system, increasing reactive oxygen species (ROS) production and reducing nitric oxide synthase activity, which ultimately leads to vascular endothelial dysfunction [38, 39]. The chronic, low-grade systemic inflammation that often accompanies aging, also plays a significant role in the development and progression of stroke [40, 41]. Consequently, the above interconnected factors jointly contribute to the development and progression of stroke.

Our study has several notable strengths. First, this is the first study to comprehensively evaluate the association between the CHG index and the risk of stroke among individuals without diabetes. This study may provide novel approaches and valuable perspectives for early detection and prompt prevention of stroke. Besides, we utilized two large nationally representative cohorts, CHARLS and NHANES, which represent Chinese and US population, enhancing the reliability of our findings. Additionally, multiple methodologies, such as multivariable-adjusted models, sensitivity and subgroup analyses, were utilized to ensure the robustness of our results.

### Limitations

Nevertheless, several limitations should be acknowledged. First, the outcome of stroke was determined based on participants’ self-reported physician diagnosis, which may introduce potential recall bias and misclassification. Second, the stroke information was relatively basic, which restricts in-depth analysis of the relationships between the CHG index and the specific stroke subtypes. Third, in this study, the number of stroke events was relatively limited for some detailed subgroup analyses, which may reduce statistical power and precision. Given that the CHARLS and NHANES cohorts represent the Chinese and US populations, respectively, the direct generalizability of our findings to other ethnic populations may be constrained. Besides, certain medications may affect serum cholesterol and glucose levels, potentially confounding the CHG index values. The absence of detailed information regarding drug classes and dosages in the current cohorts may result in residual confounding. Future prospective studies with more comprehensive pharmacological records are warranted to corroborate our findings. Finally, although our models adjusted for many covariates, residual confounding factors still cannot be entirely ruled out, which is a common problem in observational studies.

Still, in this study, we only evaluated the baseline level of the CHG index, ignoring the continuous variations of the CHG index over time. Future studies with consecutive measurements of the CHG index are warranted. Future studies are still needed to investigate the ability of CHG-derived indices, such as CHG-BMI, CHG-WC, CHG-BRI, to predict the risk of stroke. Furthermore, prospective studies exploring the association between the CHG index and stroke risk in populations beyond Chinese and US cohorts are critically important.

### Conclusions

Our study indicates that in middle-aged and elderly adults without diabetes, an elevated level of the CHG index is strongly associated with an increased risk of stroke, and a linear relationship was observed between the CHG index and the risk of stroke. These findings offer new insights into stroke prevention and provide a simple alternative for clinical practice.

## Supplementary Material

Suppl 1 Association of CHG and the risk of stroke incidence after excluding participants with missing values.

Suppl 2 Association of CHG and the risk of stroke incidence after excluding participants receiving lipid-lowering therapy.

Suppl 3 Association of CHG and the risk of stroke incidence after excluding participants receiving antihypertensive therapy.

Suppl 4 Association of CHG and the risk of stroke incidence after excluding participants with follow-up less than 2 years.

Suppl 5 Weighted baseline characteristics of participants in NHANES 2011–2018.

Suppl 6 Weighted logistic regression analysis on the association between the CHG index and stroke in NHANES 2011–2018.

Suppl 7 Receiver operating characteristic curve for the CHG index in predicting stroke incidence from NHANES 2011–2018.

## Data Availability

The datasets generated and analyzed during the current study are publicly available in the website of CHARLS (http://charls.pku.edu.cn) and NHANES (https://wwwn.cdc.gov/nchs/nhanes).

## Abbreviations

BMI: body mass index
CHG index: cholesterol–HDL–glucose index
CI: confidence interval
DBP: diastolic blood pressure
FBG: fasting blood glucose
HbA1c: hemoglobin A1c
HDL-C: high-density lipoprotein cholesterol
HR: hazard ratio
LDL-C: low-density lipoprotein cholesterol
SBP: systolic blood pressure
TC: total cholesterol
TG: triglyceride
WC: waist circumference
