# Supplementary material for: Association Between Cholesterol–HDL–Glucose Index and Stroke Risk in Middle-Aged and Elderly Adults Without Diabetes: Evidence From Two National Cohort Studies
Source: Cardiol Res. 2026 Aug 31;17(5):497–508. doi: 10.14740/cr2258 (PMC13568753; doi:10.14740/cr2258)
Supplement: Supplementary file 1 [file cr-17-05-497-s001.docx]

**Suppl 1.** Association of CHG and the risk of stroke incidence after excluding participants with missing values

|  | **Case** | **Model 1** | | **Model 2** | | **Model 3** | |
| --- | --- | --- | --- | --- | --- | --- | --- |
|  |  | **HR (95% CI)** | ***P* value** | **HR (95% CI)** | ***P* value** | **HR (95% CI)** | ***P* value** |
| CHG (per 1-unit) | 562 | 1.238 (1.037-1.479) | 0.018 | 1.242 (1.040-1.485) | 0.017 | 1.232 (1.004-1.512) | 0.046 |
| CHG quartile |  |  |  |  |  |  |  |
| Q1 | 99 | Ref |  | Ref |  | Ref |  |
| Q2 | 130 | 1.334 (1.027-1.733) | 0.031 | 1.345 (1.034-1.748) | 0.027 | 1.367 (1.046-1.788) | 0.022 |
| Q3 | 150 | 1.371 (1.064-1.769) | 0.015 | 1.370 (1.062-1.768) | 0.015 | 1.390 (1.069-1.807) | 0.014 |
| Q4 | 183 | 1.444 (1.130-1.846) | 0.003 | 1.450 (1.135-1.854) | 0.003 | 1.438 (1.106-1.870) | 0.007 |

Model 1: unadjusted.

Model 2: adjusted for age and gender.

Model 3: adjusted for age, gender, marital status, residence, education, smoking status, drinking status and hypertension.

HR: hazard ratio; CI: confidence interval.
