# Supplementary material for: Association Between Cholesterol–HDL–Glucose Index and Stroke Risk in Middle-Aged and Elderly Adults Without Diabetes: Evidence From Two National Cohort Studies
Source: Cardiol Res. 2026 Aug 31;17(5):497–508. doi: 10.14740/cr2258 (PMC13568753; doi:10.14740/cr2258)
Supplement: Supplementary file 2 [file cr-17-05-497-s002.docx]

**Suppl 2.** Association of CHG and the risk of stroke incidence after excluding participants receiving lipid-lowering therapy

|  | **Case** | **Model 1** | | **Model 2** | | **Model 3** | |
| --- | --- | --- | --- | --- | --- | --- | --- |
|  |  | **HR (95% CI)** | ***P* value** | **HR (95% CI)** | ***P* value** | **HR (95% CI)** | ***P* value** |
| CHG (per 1-unit) | 595 | 1.338 (1.120-1.599) | 0.001 | 1.343 (1.124-1.604) | 0.001 | 1.338 (1.087-1.646) | 0.006 |
| CHG quartile |  |  |  |  |  |  |  |
| Q1 | 109 | Ref |  | Ref |  | Ref |  |
| Q2 | 136 | 1.369 (1.063-1.762) | 0.015 | 1.375 (1.068-1.769) | 0.014 | 1.411 (1.089-1.830) | 0.009 |
| Q3 | 159 | 1.430 (1.120-1.825) | 0.004 | 1.432 (1.121-1.828) | 0.004 | 1.482 (1.151-1.908) | 0.002 |
| Q4 | 191 | 1.533 (1.210-1.941) | <0.001 | 1.537 (1.214-1.947) | <0.001 | 1.528 (1.184-1.971) | 0.001 |

Model 1: unadjusted.

Model 2: adjusted for age and gender.

Model 3: adjusted for age, gender, marital status, residence, education, smoking status, drinking status and hypertension.
