# Supplementary material for: Association Between Cholesterol–HDL–Glucose Index and Stroke Risk in Middle-Aged and Elderly Adults Without Diabetes: Evidence From Two National Cohort Studies
Source: Cardiol Res. 2026 Aug 31;17(5):497–508. doi: 10.14740/cr2258 (PMC13568753; doi:10.14740/cr2258)
Supplement: Supplementary file 3 [file cr-17-05-497-s003.docx]

**Suppl 3.** Association of CHG and the risk of stroke incidence after excluding participants receiving antihypertensive therapy

|  | **Case** | **Model 1** | | **Model 2** | | **Model 3** | |
| --- | --- | --- | --- | --- | --- | --- | --- |
|  |  | **HR (95% CI)** | ***P* value** | **HR (95% CI)** | ***P* value** | **HR (95% CI)** | ***P* value** |
| CHG (per 1-unit) | 453 | 1.305 (1.058-1.611) | 0.013 | 1.325 (1.072-1.637) | 0.009 | 1.333 (1.042-1.706) | 0.022 |
| CHG quartile |  |  |  |  |  |  |  |
| Q1 | 88 | Ref |  | Ref |  | Ref |  |
| Q2 | 96 | 1.354 (1.013-1.809) | 0.041 | 1.373 (1.027-1.835) | 0.033 | 1.391 (1.031-1.877) | 0.031 |
| Q3 | 130 | 1.365 (1.041-1.790) | 0.025 | 1.366 (1.041-1.791) | 0.024 | 1.385 (1.047-1.832) | 0.022 |
| Q4 | 139 | 1.425 (1.091-1.863) | 0.009 | 1.429 (1.093-1.868) | 0.009 | 1.417 (1.063-1.889) | 0.018 |

Model 1: unadjusted.

Model 2: adjusted for age and gender.

Model 3: adjusted for age, gender, marital status, residence, education, smoking status, drinking status and hypertension.
