# Supplementary material for: Association Between Cholesterol–HDL–Glucose Index and Stroke Risk in Middle-Aged and Elderly Adults Without Diabetes: Evidence From Two National Cohort Studies
Source: Cardiol Res. 2026 Aug 31;17(5):497–508. doi: 10.14740/cr2258 (PMC13568753; doi:10.14740/cr2258)
Supplement: Supplementary file 4 [file cr-17-05-497-s004.docx]

**Suppl 4.** Association of CHG and the risk of stroke incidence after excluding participants with follow-up less than 2 years

|  | **Case** | **Model 1** | | **Model 2** | | **Model 3** | |
| --- | --- | --- | --- | --- | --- | --- | --- |
|  |  | **HR (95% CI)** | ***P* value** | **HR (95% CI)** | ***P* value** | **HR (95% CI)** | ***P* value** |
| CHG (per 1-unit) | 594 | 1.221 (1.025-1.453) | 0.025 | 1.224 (1.028-1.458) | 0.024 | 1.227 (1.005-1.498) | 0.044 |
| CHG quartile |  |  |  |  |  |  |  |
| Q1 | 111 | Ref |  | Ref |  | Ref |  |
| Q2 | 136 | 1.334 (1.037-1.714) | 0.025 | 1.339 (1.042-1.722) | 0.023 | 1.346 (1.040-1.742) | 0.024 |
| Q3 | 161 | 1.356 (1.064-1.728) | 0.014 | 1.357 (1.064-1.729) | 0.014 | 1.387 (1.080-1.780) | 0.010 |
| Q4 | 186 | 1.395 (1.102-1.766) | 0.006 | 1.401 (1.107-1.773) | 0.005 | 1.395 (1.084-1.795) | 0.010 |

Model 1: unadjusted.

Model 2: adjusted for age and gender.

Model 3: adjusted for age, gender, marital status, residence, education, smoking status, drinking status and hypertension.
