# Supplementary material for: Association Between Cholesterol–HDL–Glucose Index and Stroke Risk in Middle-Aged and Elderly Adults Without Diabetes: Evidence From Two National Cohort Studies
Source: Cardiol Res. 2026 Aug 31;17(5):497–508. doi: 10.14740/cr2258 (PMC13568753; doi:10.14740/cr2258)
Supplement: Supplementary file 5 [file cr-17-05-497-s005.docx]

**Suppl 5.** Weighted baseline characteristics of participants in NHANES 2011‒2018

| **Characteristics** | **Overall** | **Quartiles of CHG** | | | | ***P* value** |
| --- | --- | --- | --- | --- | --- | --- |
|  |  | **Q1** | **Q2** | **Q3** | **Q4** |  |
| Number of participants | 5057 | 1288 | 1295 | 1220 | 1254 |  |
| Age, years | 61.57 (61.08-62.06) | 62.25 (61.33-63.18) | 62.41 (61.60-63.21) | 61.05 (60.37-61.73) | 60.44 (59.80-61.07) | <0.001 |
| Gender, % |  |  |  |  |  | <0.001 |
| Male | 45.92 (44.18-47.65) | 33.39 (28.72-38.06) | 41.95 (38.40-45.49) | 50.09 (46.52-53.65) | 60.05 (56.15-63.95) |  |
| Female | 54.09 (52.35-55.82) | 66.61 (61.94-71.28) | 58.05 (54.51-61.60) | 49.91 (46.35-53.48) | 39.95 (36.06-43.85) |  |
| Race, % |  |  |  |  |  | <0.001 |
| Mexican American | 5.33 (3.96-6.70) | 3.40 (2.20-4.60) | 4.89 (3.57-6.22) | 5.79 (4.03-7.54) | 7.53 (5.44-9.61) |  |
| Other Hispanic | 4.65 (3.55-5.76) | 2.84 (1.81-3.87) | 4.22 (3.01-5.43) | 5.91 (4.20-7.61) | 5.87 (4.37-7.37) |  |
| Non-Hispanic White | 73.20 (69.93-76.47) | 75.57 (72.00-79.13) | 71.81 (67.79-75.83) | 73.84 (69.71-77.97) | 71.28 (67.00-75.57) |  |
| Non-Hispanic Black | 9.35 (7.61-11.09) | 11.61 (9.16-14.06) | 10.67 (8.54-12.79) | 6.71 (5.09-8.32) | 8.16 (6.32-10.00) |  |
| Other races | 7.46 (6.15-8.78) | 6.58 (5.02-8.13) | 8.42 (6.14-10.70) | 7.76 (5.92-9.60) | 7.16 (4.91-9.40) |  |
| Marital status, % |  |  |  |  |  | 0.235 |
| Married | 71.93 (69.41-74.46) | 74.01 (70.11-77.90) | 71.76 (67.80-75.72) | 72.89 (68.82-76.95) | 68.73 (64.41-73.06) |  |
| Others | 28.07 (25.54-30.59) | 25.99 (22.10-29.89) | 28.24 (24.28-32.20) | 27.11 (23.05-31.18) | 31.27 (26.94-35.59) |  |
| Education, % |  |  |  |  |  | <0.001 |
| Below high school | 5.81 (4.84-6.78) | 4.79 (3.47-6.12) | 6.15 (4.51-7.78) | 5.58 (4.20-6.96) | 6.86 (5.35-8.37) |  |
| High school/GED | 32.68 (30.30-35.05) | 27.92 (24.52-31.32) | 31.17 (27.49-34.86) | 34.97 (31.21-38.73) | 37.29 (33.03-41.55) |  |
| Above high school | 61.51 (58.80-64.22) | 67.29 (63.58-71.00) | 62.68 (58.61-66.75) | 59.45 (55.61-63.30) | 55.85 (51.43-60.27) |  |
| Smoking, % | 48.31 (45.79-50.84) | 44.22 (40.19-48.25) | 46.20 (41.88-50.51) | 47.95 (44.54-51.36) | 55.62 (51.01-60.23) | <0.001 |
| Drinking, % | 69.31 (66.53-72.09) | 73.58 (70.11-77.04) | 66.67 (61.99-71.34) | 67.80 (63.43-72.17) | 68.81 (65.09-72.53) | 0.033 |
| Hypertension, % | 48.27 (45.84-50.71) | 41.33 (37.06-45.61) | 46.75 (42.19-51.31) | 49.76 (45.70-53.82) | 56.26 (52.65-59.87) | <0.001 |
| Medication, % |  |  |  |  |  |  |
| Lipid-lowering | 33.50 (31.66-35.35) | 33.28 (29.37-37.18) | 35.65 (32.22-39.08) | 31.02 (27.54-34.49) | 34.12 (29.85-38.38) | 0.395 |
| Antihypertensive | 40.62 (38.18-43.07) | 34.92 (31.27-38.57) | 40.58 (36.16-45.01) | 41.02 (37.01-45.02) | 46.77 (43.13-50.42) | <0.001 |
| WC, cm | 101.72 (100.91-102.54) | 93.99 (92.56-95.41) | 101.28 (100.13-102.44) | 103.94 (102.59-105.28) | 108.61 (107.30-109.92) | <0.001 |
| BMI, kg/m^2^ | 29.22 (28.89-29.55) | 26.48 (26.01-26.96) | 29.24 (28.75-29.74) | 29.94 (29.28-30.60) | 31.57 (31.05-32.09) | <0.001 |
| SBP, mmHg | 127.16 (126.25-128.08) | 125.22 (123.49-126.94) | 127.17 (125.70-128.65) | 127.31 (126.36-128.26) | 129.20 (127.31-131.10) | 0.024 |
| DBP, mmHg | 70.12 (69.43-70.82) | 68.50 (67.52-69.47) | 70.12 (68.94-71.30) | 70.62 (69.68-71.56) | 71.45 (70.16-72.74) | <0.001 |
| TC, mg/dL | 194.14 (192.32-195.96) | 183.95 (180.99-186.92) | 188.95 (186.19-191.71) | 197.17 (193.28-201.05) | 208.09 (204.53-211.64) | <0.001 |
| TG, mg/dL | 116.28 (112.20-120.36) | 71.92 (68.93-74.92) | 99.66 (96.44-102.88) | 121.18 (117.50-124.86) | 178.89 (165.05-192.73) | <0.001 |
| HDL-C, mg/dL | 57.68 (56.73-58.63) | 74.70 (72.77-76.62) | 58.89 (57.88-59.90) | 51.35 (50.52-52.18) | 43.58 (42.75-44.41) | <0.001 |
| LDL-C, mg/dL | 113.33 (111.88-114.78) | 94.77 (92.61-96.93) | 110.10 (107.90-112.31) | 121.61 (118.46-124.76) | 129.50 (126.24-132.76) | <0.001 |
| HbA1c, % | 5.75 (5.71-5.78) | 5.53 (5.49-5.56) | 5.66 (5.62-5.69) | 5.79 (5.74-5.84) | 5.91 (5.85-5.97) | <0.001 |
| FBG, mg/dL | 106.52 (105.41-107.62) | 97.21 (96.37-98.04) | 104.30 (103.35-105.24) | 108.45 (107.14-109.77) | 115.24 (112.22-118.25) | <0.001 |
| Stroke, % | 5.81 (5.01-6.60) | 3.35 (2.41-4.30) | 5.80 (4.31-7.29) | 6.02 (4.56-7.49) | 8.39 (6.69-10.08) | <0.001 |

Continuous variables are presented as weighted means and 95% confidence interval. Categorical variables are presented as weighted percentage and 95% confidence interval.

WC: waist circumference; BMI: body mass index; SBP: systolic blood pressure; DBP: diastolic blood pressure; TC: total cholesterol; TG: triglyceride; HDL-C: high-density lipoprotein cholesterol; LDL-C: low-density lipoprotein cholesterol; HbA1c: hemoglobin A1c; FBG: fasting blood glucose; GED: general educational development.
