# Supplementary material for: Association Between Cholesterol–HDL–Glucose Index and Stroke Risk in Middle-Aged and Elderly Adults Without Diabetes: Evidence From Two National Cohort Studies
Source: Cardiol Res. 2026 Aug 31;17(5):497–508. doi: 10.14740/cr2258 (PMC13568753; doi:10.14740/cr2258)
Supplement: Supplementary file 6 [file cr-17-05-497-s006.docx]

**Suppl 6.** Weighted logistic regression analysis on the association between the CHG index and stroke in NHANES 2011‒2018

| **CHG index** | **Case** | **Model 1** | | **Model 2** | | **Model 3** | |
| --- | --- | --- | --- | --- | --- | --- | --- |
|  |  | **OR (95% CI)** | ***P* value** | **OR (95% CI)** | ***P* value** | **OR (95% CI)** | ***P* value** |
| Continuous | 366 | 2.853 (1.851-4.398) | <0.001 | 3.497 (2.281-5.361) | <0.001 | 2.610 (1.729-3.937) | <0.001 |
| Quartiles |  |  |  |  |  |  |  |
| Q1 | 55 | Ref |  | Ref |  | Ref |  |
| Q2 | 88 | 1.774 (1.235-2.548) | 0.002 | 1.830 (1.261-2.656) | 0.002 | 1.738 (1.179-2.562) | 0.006 |
| Q3 | 108 | 1.848 (1.220-2.800) | 0.004 | 2.122 (1.407-3.201) | 0.001 | 1.901 (1.234-2.931) | 0.004 |
| Q4 | 115 | 2.639 (1.802-3.865) | <0.001 | 3.247 (2.207-4.778) | <0.001 | 2.467 (1.685-3.613) | <0.001 |

Model 1: unadjusted.

Model 2: adjusted for age and gender.

Model 3: adjusted for age, gender, marital status, education, smoking status, drinking status and hypertension.

OR: odds ratio; CI: confidence interval.
