# Supplementary material for: Association Between Cholesterol–HDL–Glucose Index and Stroke Risk in Middle-Aged and Elderly Adults Without Diabetes: Evidence From Two National Cohort Studies
Source: Cardiol Res. 2026 Aug 31;17(5):497–508. doi: 10.14740/cr2258 (PMC13568753; doi:10.14740/cr2258)
Supplement: Supplementary file 7 [file cr-17-05-497-s007.docx]

**Suppl 7.** Receiver operating characteristic curve for the CHG index in predicting stroke incidence from NHANES 2011‒2018.
